# Synthesis and Characterization of Vanadium Nitride/Carbon Nanocomposites

**DOI:** 10.3390/ijms25136952

**Published:** 2024-06-25

**Authors:** Helia Magali Morales, Horacio Vieyra, David A. Sanchez, Elizabeth M. Fletes, Michael Odlyzko, Timothy P. Lodge, Victoria Padilla-Gainza, Mataz Alcoutlabi, Jason G. Parsons

**Affiliations:** 1 School of Integrative Biological and Chemical Sciences, University of Texas Rio Grande Valley, 1 West University Blvd., Brownsville, TX 78521, USA; helia.morales@utrgv.edu; 2 Escuela de Ingeniería y Ciencias, Tecnológico de Monterrey, Av E. Garza Sada 2501, Monterrey 64849, NL, Mexico; h.vieyra@tec.mx; 3 Department of Mechanical Engineering, University of Texas Rio Grande Valley, 1201 West University Dr., Edinburg, TX 78539, USA; david.sanchez11@utrgv.edu (D.A.S.); elizabeth.fletes01@utrgv.edu (E.M.F.); victoria.padilla@utrgv.edu (V.P.-G.); 4 Characterization Facility, College of Science and Engineering, 55 Shepherd Laboratories, University of Minnesota, 100 Union Street SE, Minneapolis, MN 55455, USA; odlyz003@umn.edu; 5 Department of Chemical Engineering and Materials Science, University of Minnesota, 421 Washington Avenue SE, Minneapolis, MN 55455, USA; lodge@umn.edu; 6 Department of Chemistry, University of Minnesota, 207 Pleasant Street SE, Minneapolis, MN 55455, USA; 7 School of Earth Environmental and Marine Sciences, University of Texas Rio Grande Valley, 1 West University Blvd., Brownsville, TX 78521, USA

**Keywords:** vanadium nitride/carbon nanocomposite, sol-gel method, thermal degradation method, energy storage

## Abstract

The present work focuses on the synthesis of a vanadium nitride (VN)/carbon nanocomposite material via the thermal decomposition of vanadyl phthalocyanine (VOPC). The morphology and chemical structure of the synthesized compounds were characterized using scanning electron microscopy (SEM), transmission electron microscopy (TEM), energy dispersive spectroscopy (EDS), Fourier transformed infrared spectroscopy (FTIR), X-ray diffraction (XRD), and X-ray photoemission spectroscopy (XPS). The successful syntheses of the VOPC and non-metalated phthalocyanine (H_2_PC) precursors were confirmed using FTIR and XRD. The VN particles present a needle-like morphology in the VN synthesized by the sol-gel method. The morphology of the VN/C composite material exhibited small clusters of VN particles. The XRD analysis of the thermally decomposed VOPC indicated a mixture of amorphous carbon and VN nanoparticles (VN(TD)) with a cubic structure in the space group FM-3M consistent with that of VN. The XPS results confirmed the presence of V(III)-N bonds in the resultant material, indicating the formation of a VN/C nanocomposite. The VN/C nanocomposite synthesized through thermal decomposition exhibited a high carbon content and a cluster-like distribution of VN particles. The VN/C nanocomposite was used as an anode material in LIBs, which delivered a specific capacity of 307 mAh g^−1^ after 100 cycles and an excellent Coulombic efficiency of 99.8 at the 100th cycle.

## 1. Introduction

In recent years, metal nitrides have garnered interest for applications in energy storage, energy conversion, catalysts, and photocatalysis [1,2,3]. Metal nitrides outperform metal oxides, sulfides, and carbides as substitute materials for many applications in which noble metals are otherwise heavily used, serving as potential low-cost alternatives to materials such as silver and gold [4]. Metal nitrides exhibit high melting points, high hardness, high stiffness, wide band gaps, high metallic electrical conductivity, and good thermal and chemical stability [2,4,5]. VN has been synthesized through diverse methods, including combustion reactions [6], sol-gel [7], magnetron sputtering [8], mechano-synthesis [9], metathesis [10,11], nitridation [12], ammonolysis [13], carbothermal reduction-nitridation [14], electrochemical deposition [15], vapor deposition [16], and thermal decomposition [17].

Efforts to improve the electrochemical performance of nitrides have led to the development of nitride/carbon and metal oxide/carbon composites [18,19,20] with high electrical conductivities, surface areas, and favorable environmental impacts [5,21,22]. Fischer et al. [22] synthesized metal nitride nanoparticles in mesoporous graphitic carbon nitride (C_3_N_4_). The C_3_N_4_ was prepared using cyanamide and silica nanoparticles, with the latter influencing the size distribution of the nitride nanoparticles. Metal oxide nanoparticles were produced in the silica pores by sol-gel reaction and subsequent thermal decomposition at 800 °C under an inert atmosphere converted metal oxides into corresponding metal nitrides. Cui et al. [23] prepared a TiVN/carbon composite with different V/Ti ratios using a synthesis method developed by Fischer et al. [22]. A composite anode with a 3:1 V/Ti ratio exhibited the highest reversible capacity, 678 mAh g^−1^ at 74.4 mAg^−1^. Sun et al. [24] prepared a porous VN nanoribbon/graphene composite for a lithium–sulfur cathode material. The graphene facilitated the transport of electrons and lithium ions, while the vanadium nitride served as a chemical anchor and catalyzed the redox reaction. Charge and discharge capacities reached 1471 mAh g^−1^ and 1252 mAh g^−1^ after 100 cycles at 0.2 C, respectively. Li et al. [25] synthesized a free-standing flexible electrode of vanadium nitride within an N-doped carbon/graphene nanostructure. V_2_O_5_ nanowires were prepared using a hydrothermal process, which were then coated with polypyrrole through a polymerization reaction. The nanowires were washed and annealed at 550 °C in an ammonia atmosphere, resulting in a VN/carbon composite material. An electrode was prepared by mixing the VN/carbon composite material with graphene and D-D-dimethylformamide. The VN/C composite anode delivered a capacity of 524 Ah g^−1^ after 200 cycles and a current density of 5 mA cm^−2^. Qi et al. [26] reported the synthesis of porous metal nitride–carbon composites via liquid–crystalline templating in liquid NH_3_. The resultant products were characterized by different techniques, and it was found that the porosity, surface area and morphology of the composites depended on reactant stoichiometry. 

Carbon-based composites can also be prepared from metal–organic compounds serving as sacrificial templates, enabling the fabrication of metallic nanoparticles dispersed in a carbon matrix [27]. The selection of appropriate starting materials enables control of the resulting structure, producing materials with high porosity, high surface area, and good electrochemical properties. Metal-substituted phthalocyanines are well-known conjugated macrocycles which can be used as precursors to produce metal nitride/amorphous carbon composites [28].

In the present work, a one-step pyrolysis method was developed to prepare VN/C nanocomposites through the thermal decomposition of vanadyl phthalocyanine (VOPC). The current investigation is aimed at developing a one-molecule precursor with long-term stability to synthesize VN/C composite materials for use as anode materials in LIBs. In addition, the current study is also an attempt to develop a composite material without the formation of particle segregation, surface aggregation, or island formation caused by miscibility or other chemical characteristics, which prevent homogeneity in composites. For comparison purposes, VN was also synthesized using the sol-gel method through the reaction of an alkoxide with urea. The structure and morphology of the resultant materials were characterized by X-ray diffraction (XRD), thermogravimetric analysis (TGA), Fourier transform infrared spectroscopy (FTIR), scanning electron microscopy (SEM), energy dispersive X-ray spectrometry (EDS), transmission electron microscopy (TEM), and X-ray photoelectron spectroscopy (XPS). The VN/carbon nanocomposite was used as an anode in Li-ion half cells, and its electrochemical performance was evaluated using cyclic voltammetry (CV) and galvanostatic charge–discharge experiments.

## 2. Results and Discussion

### 2.1. FTIR

**Figure 1:**
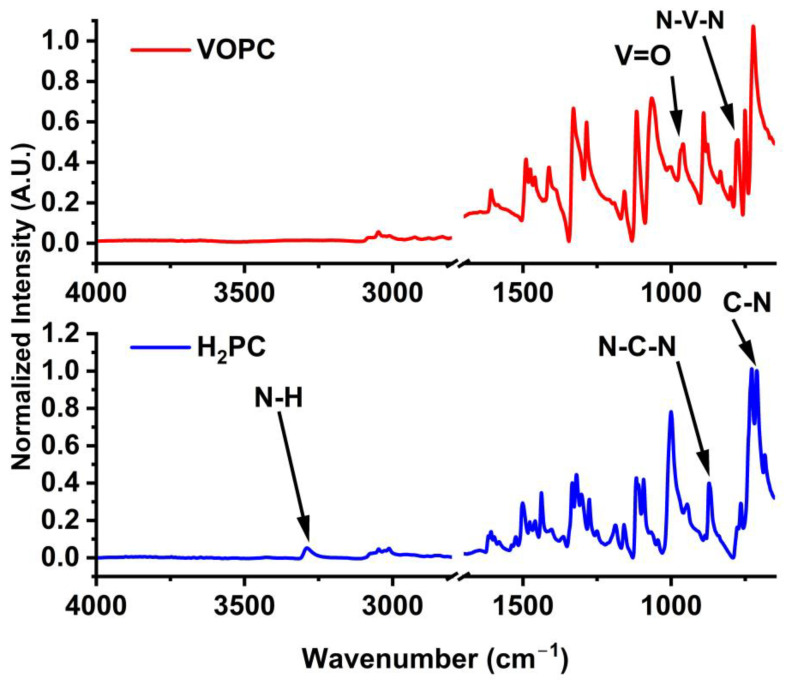
FTIR spectra of synthesized VOPC (**top**) and H_2_PC (**bottom**).

FTIR spectra collected for the H_2_PC and VOPC compounds are shown in Figure 1. The identified peaks are summarized in Table 1.

The FTIR spectra of the synthesized compounds display the expected characteristic bands of metal-free phthalocyanine (H_2_PC) and VOPC. The synthesis of the VOPC was confirmed by the band at 953 cm^−1^, which indicates the presence of V=O [32]. The N-H stretches observed at 3282 cm^−1^ in the H_2_PC compound are missing in the VOPC complex, indicating the loss of N-H bonding due to the formation of V-N bonds. The appearance of the C-N stretches in the macrocycle rings at lower wavenumbers further confirms the formation of V-N bonds, which account for the shift to a lower wave number. FTIR characterization confirms the synthesis of both H_2_PC and VOPC, in which all the characteristic peaks of each compound were observed in the spectra.

### 2.2. SEM/EDS

**Figure 2:**
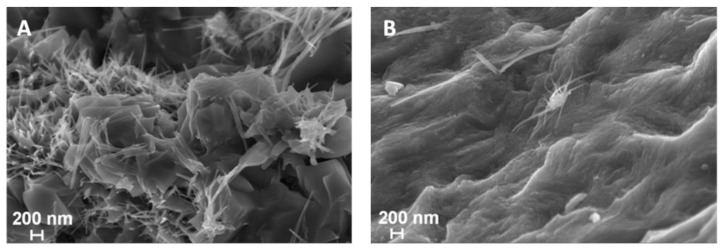
SEM images of (**A**) VN(SG) and (**B**) VN(TD).

The morphologies of the synthesized complexes were investigated using SEM. The morphology of VN(SG) is shown in Figure 2A, presenting a nanostructured architecture consisting of both needles and some small platelets. The needles in Figure 2A show lengths ranging from 200 to 400 nm and thicknesses between 10 and 20 nm. The plates are visible in the VN sample (Figure 2A) and show sizes up to 1μm lengths and a 5–10 nm thickness. The formation of the structures in the samples probably was due to the thermal decomposition of the alkoxide–urea precursor leading to the specific growth. This was due to the different decomposition temperature of the precursor components and the higher reaction temperature. The morphology of the VN(TD) (Figure 2B) shows some small structures embedded in the undulating surface, while some larger needle-like structures are observed on the surface of the sample. The bulk of the sample should be carbon, which should be the undulating surface, from the high-temperature decomposition of the VOPC precursor.

**Figure 3:**
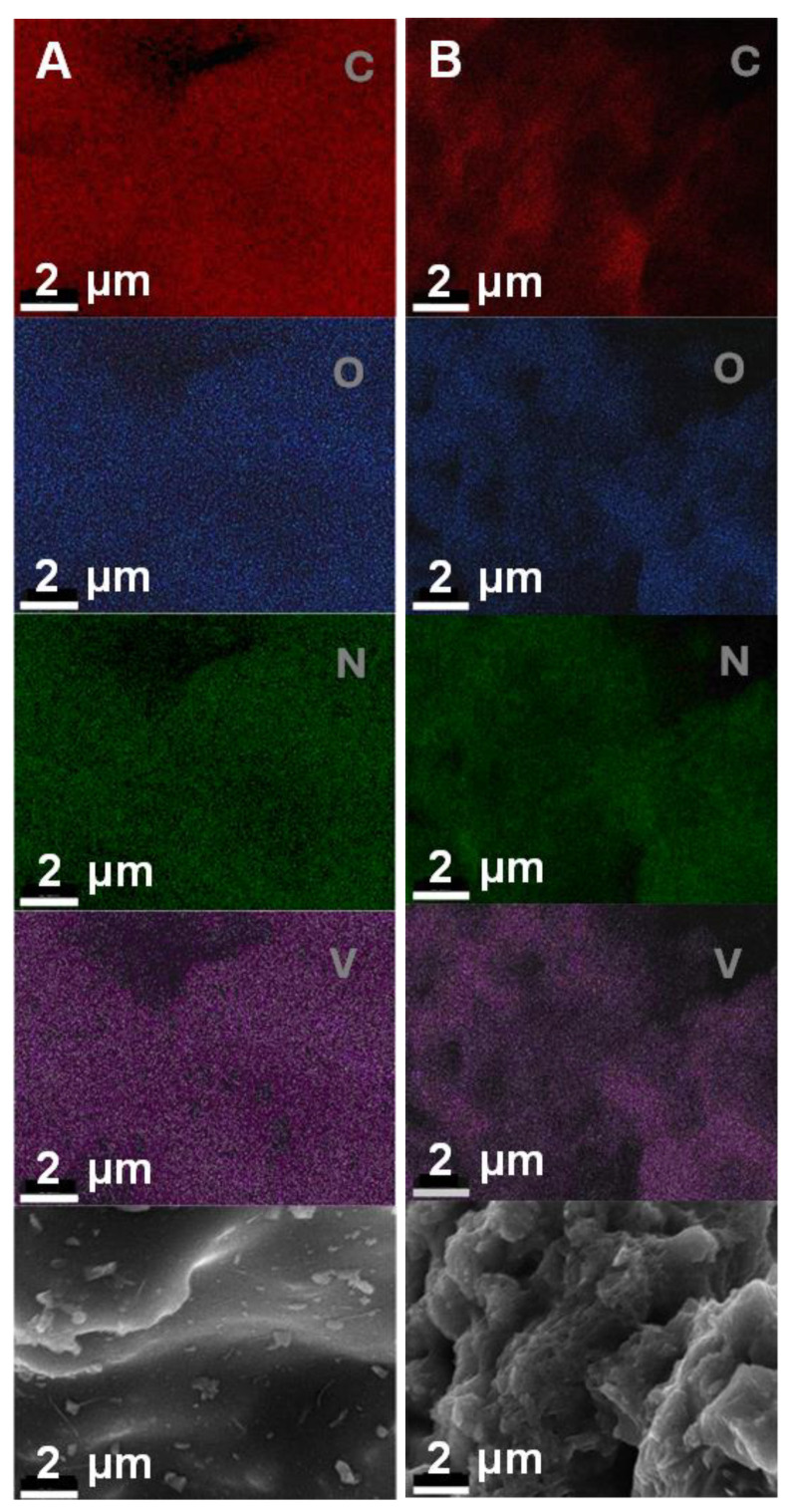
EDS elemental mapping of (**A**) VN(TD) and (**B**) VN(SG). The bottom panes show the secondary electron images.

Figure 3 summarizes the EDS elemental mapping of the vanadium nitrides synthesized through the sol-gel method, VN(SG) (A), and thermally decomposed metal-substituted phthalocyanine, VN(TD) (B). For both the VN(SG) and VN(TD) samples, there is an excellent correlation in the elemental mappings between all the elements in the samples. Both the V and N appear to be co-located throughout the samples, as well as the O, which indicates that these elements are in a common compound. The EDS data show that a uniform distribution of the constituent elements in both samples was achieved.

Table 2 summarizes the elemental composition of the VOPC, VN(SG), and VN(TD) materials. All samples contained detectable C, N, O, and V. The VOPC precursor had the lowest proportion of V present in the sample. The VN(TD) product exhibited an average composition of 9% V, an approximately 6-fold increase, which was expected due to sample conversion in the thermal decomposition reaction. The thermal decomposition would cause the loss of small molecules such as H_2_ or small organic molecules and would increase the relative amount of VN in the sample compared to the starting material. The VN(SG) sample was measured as approximately 34% V, the highest of all three samples, as would be expected. The higher amount of VN in the sample compared to the other samples was expected because the sample should be relatively pure VN with very little amorphous carbon present.

### 2.3. TEM

**Figure 4:**
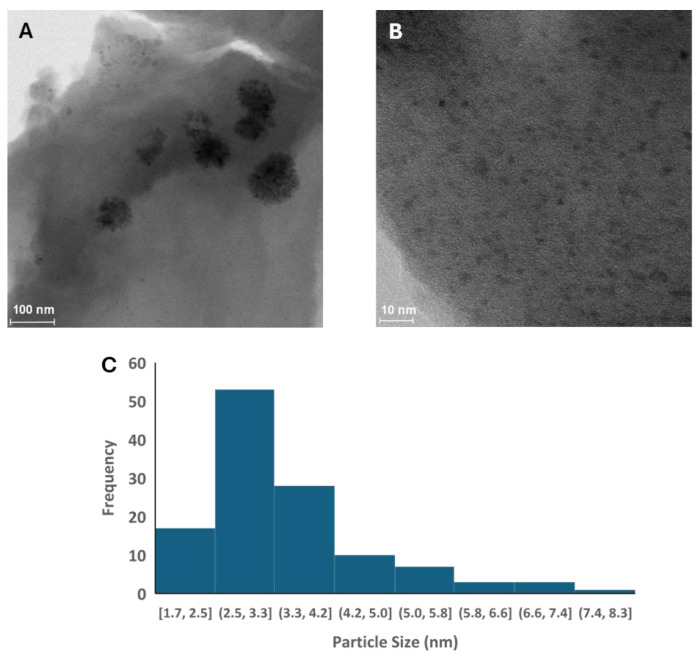
High-resolution TEM images of VN(TD) (**A**,**B**) and particle size distribution (**C**).

Figure 4 shows high-resolution TEM images of the VN(TD). It is shown in Figure 4A that the VN appears as clusters embedded in the organic matrix; there are no needle-like structures present. The clusters of VN nanoparticles appear as round fine nanoparticles smaller than 10 nm in diameter. Figure 4B shows a higher magnification of one of the clusters, confirming that there is a relatively uniform distribution of VN nanocrystals within the clusters. Figure 4C shows the particle size distribution of the VN within the clusters and the VN scattered throughout samples. As can be seen in Figure 4C, most of the counted particles did fall within the 2.5–3.3 nm range. The average particle size was determined to be approximately 3.5 ± 1.2 nm. The average particle size was based on the measurement of 122 particles in the sample.

### 2.4. XRD

**Figure 5:**
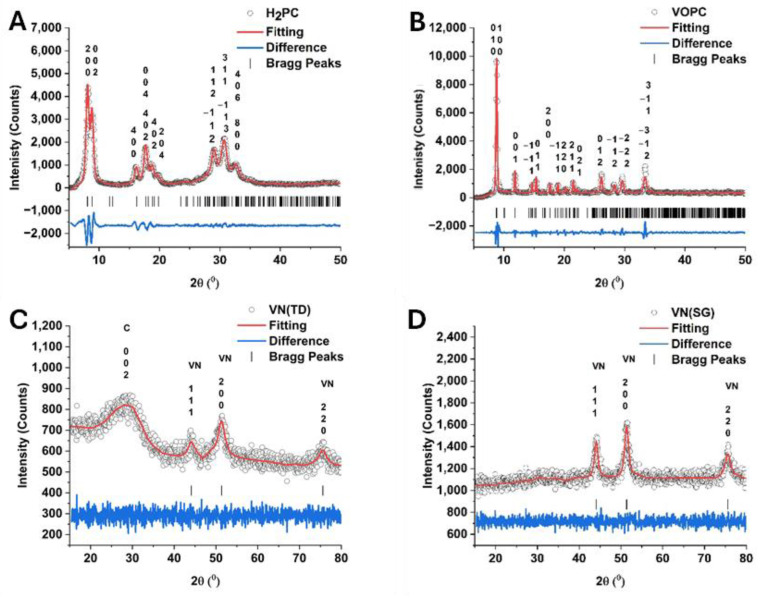
XRD patterns of (**A**) H_2_PC, (**B**) VOPC, (**C**) VN(TD), and (**D**) VN (SG).

Figure 5 panels A, B, C, and D present the XRD powder diffraction patterns for H_2_PC VOPC, VN(TD), and VN(SG), respectively. Table 3 summarizes the Le Bail fitting results for each compound, which shows χ^2^ values below 5, indicating a good agreement between the fits and the experimental data [35,36].

The H_2_PC complex was found to fit well with the results reported in the literature for a crystal structure with a C_2_/N space group with a large lattice. The H_2_PC complex showed slightly shorter lattice parameters in the current paper compared to those reported in the literature; however, they are within the range of error for fitting. The VOPC complex was determined to be in the triclinic P-1 space group with lattice parameters that are consistent with the literature [38]. The VN(SG) and VN(TD) were observed to have a cubic FM-3M lattice with *a* = *b* = *c* = 4.13 Å and an χ^2^ of 1.02 and 1.11, respectively. The fitting shows that there is a high degree agreement between the synthesized samples and the literature [39]. The diffraction pattern of the VN(TD) shows the presence of a broad weak peak located at around 29° in 2θ, indicating the presence of amorphous carbon in the sample. The weak diffraction peak observed in the sample corresponds to the 002 plane observed in amorphous carbon samples. The XRD data indicate the synthesis of a hybrid VN/carbon material as the result of the thermal decomposition of the metal-substituted phthalocyanine. Using Scherrer’s equation, the average crystallite sizes of the nanoparticles were determined to be 5.2 ± 1.0 nm and 13.6 ± 1.5 nm for the VN(TD) and the VN(SG) samples, respectively. The average size of the VN(TD) particles determined from the TEM images was approximately 3–4 nm, which agrees with the average crystallite size determined from the XRD. Both techniques show the presence of VN and amorphous carbon in the VN(TD) sample, confirming the desired synthesis of a VN/carbon nanocomposite. The VN(SG) synthesis did not show the presence of the C 002 peak in the diffraction pattern, indicating that either particularly amorphous carbon was present or a very low concentration of carbon was observed. Which means that it is more than likely a composite material was not formed.

### 2.5. XPS

**Figure 6:**
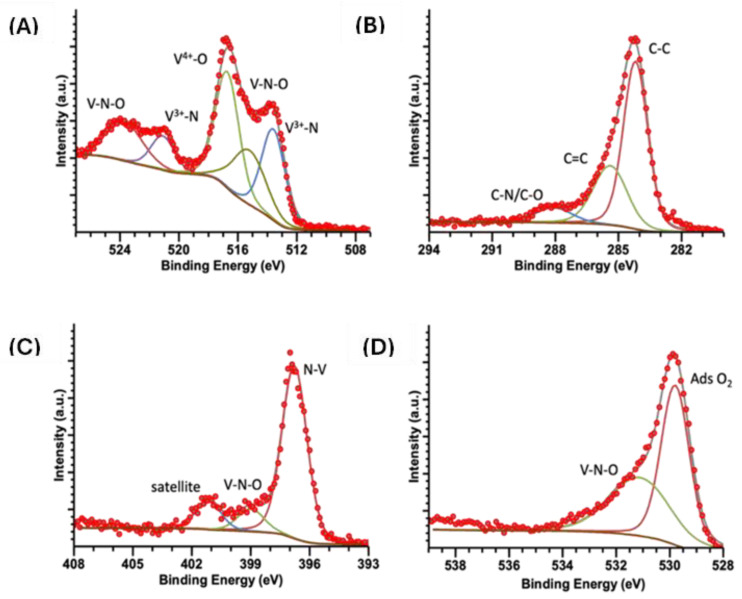
XPS spectra for VN(SG) (**A**) V 2p, (**B**) C 1s, (**C**) N 1s, (**D**) O 1s.

**Figure 7:**
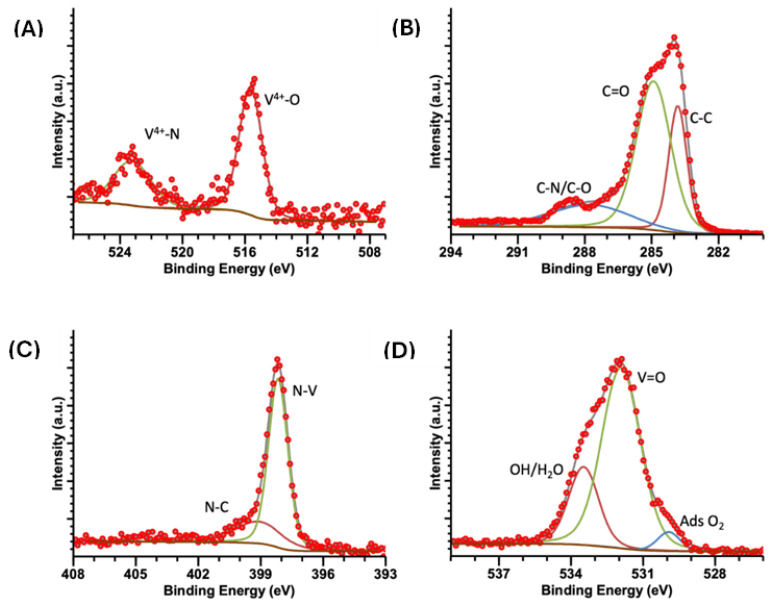
XPS spectra of VOPC (**A**) V 2p, (**B**) C 1s, (**C**) N 1s, (**D**) O 1s.

**Figure 8:**
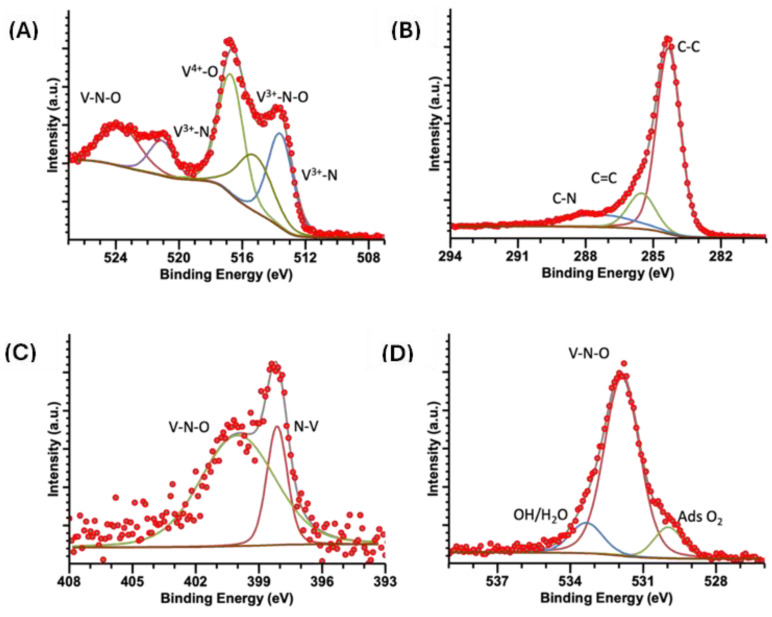
XPS spectra of VN(TD) (**A**) V 2p, (**B**) C 1s, (**C**) N 1s, (**D**) O 1s.

Figure 6, Figure 7 and Figure 8 show high-resolution XPS spectra for the vanadium compounds VOPC, VN(SG), and VN(TD). Figure 6A displays the VN(SG) sample’s spectra, which showed two peaks representing the V 2p_3/2_ and V 2p_1/2_ electronic transitions. The V 2p_3/2_ peak was deconvolved into three individual peaks at 513.6, 515.3, and 516.7 eV, which is representative of V^3+^ bound to an N, VN-O coordination, and V-O(V^4+^) coordination environments [16]. The V 2p_1/2_ peak was deconvolved into two individual peaks at 521.1 and 523.7, representing V^3+^ bound to a N and a VN-O coordination environment [40]. Figure 7A shows the V 2p spectrum for the synthesized VOPC sample. The spectrum consisted of two peaks at 515.6 and 523.2 eV, representing the V 2p_3/2_ and the V 2p_1/2_, respectively. The energies of the two peaks are consistent with the values reported for V^4+^ bound to a nitrogen ligand and an oxygen ligand [41]. The same coordination environments were observed in the VN(TD) and VN(SG) samples. Figure 8A shows the V 2p spectrum for the VN(TD) sample. The V 2p_3/2_ peak was deconvolved into three peaks at 513.2, 515.0, and 516.4 eV, representing V^3+^ bound to an N, VN-O coordination, and V-O (V^4+^) coordination environments. The V 2p_1/2_ peak was deconvolved into two individual peaks at 521.0 and 523.4 eV, representing V^3+^ bound to an N, VN-O coordination, and V-O (V^4+^).

Figure 6B shows the C 1s XPS spectra for the VN(SG) sample, which show the presence of a peak deconvolved into three peaks at 284.2, 285.4, and 288.1 eV. The peaks represent C in the C-C, C=C, and C-N/C-O environments [42,43]. Figure 7B shows the high-resolution XPS spectra for the C 1s region of the synthesized VOPC sample. The C 1s peak was determined to consist of three individual coordination environments located at 283.8, 284.9, and 287.9 eV, which corresponded to carbon in the C-C, C=C, and C-N environments, which are consistent with the carbon environments found in VOPC [41]. Figure 8B shows the C 1s peak for the VN(TD) sample. The VN(TD) and VN(SG) binding environments were identical, with three peaks located at 284.3, 285.5, and 287.4 eV, which are consistent with the peaks from amorphous carbon [41,43].

The N 1s XPS spectrum for the VN(SG) sample, shown in Figure 6C, consisted of one peak, which was deconvolved into three individual peaks located at 396.8, 399.0, and 401.2 eV, where the energies corresponded to N bound to V, V-N-O, and a satellite peak [44]. Figure 7C shows the XPS spectrum for the N 1s in the VOPC complex, which consisted of two binding environments observed at energies of 398.2 and 398.9 eV. The 398.2 and 398.9 eV binding energies represent the N-V coordination and C-N binding environments, respectively [45]. Figure 8C shows the N 1s XPS spectrum for the VN(TD) sample and indicates one peak, which was deconvolved into two individual peaks with binding energies of 398.1 and 400.0 eV. The peak located at 398.1 eV represents N bound to the V ion [46], while the peak located at 400.0 eV corresponds to N in a V-N-O coordination environment [41]. 

The O 1s spectrum for the VN(SG) sample is shown in Figure 6D. The O 1s peak for the VN sample was determined to consist of two peaks located at 529.8 and 531.2 eV. The peak located at 529.8 eV is attributed to adsorbed oxygen in the sample [47], while the peak located at 531.2 is attributed to the formation of V-N-O on the surface of the VN [41]. The O 1s XPS spectrum for the VOPC sample is shown in Figure 7D, indicating one peak consisting of three individual peaks located at 529.9, 531.9, and 533.5 eV. The peak located at 529.9 eV corresponds to adsorbed oxygen in the sample [47], while the peak located at 531.9 eV is attributed to the V=O bond [48]. The peak at 533.5 corresponds to water/hydroxide in the sample. The VN(TD) sample spectra are shown in Figure 8D. The O 1s peak for the VN formed through the thermal decomposition of VOPC consisted of three peaks at 529.9, 531.9 and 533.3 eV. These peaks are attributed to the presence of adsorbed oxygen, hydroxide/water adsorbed to the compound, and V-N-O on the surface of the compound, respectively [46].

The XPS data revealed that the VN(SG) and VN(TD) have similar binding environments, with no evidence of vanadium carbide. This confirms the successful synthesis of the VN via the sol-gel method and a VN/carbon composite resulting from the thermal decomposition method. The features of the XPS data are summarized below in Table 4, including the bonding interactions associated with each peak.

### 2.6. Cyclic Voltammetry

**Figure 9:**
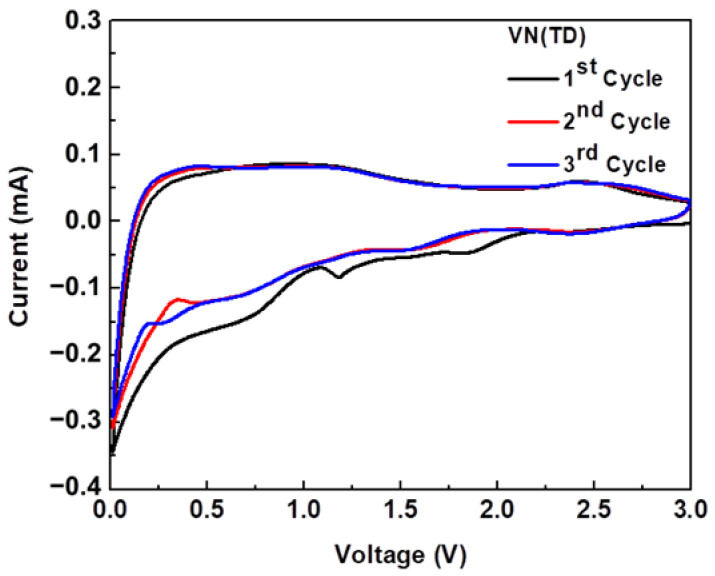
Cyclic voltammetry curves of VN (TD)/C composite electrode synthesized by the thermal decomposition of vanadyl phthalocyanine.

To understand the physical interaction of Li^+^ with the VN(TD) electrode material, cyclic voltammetry (CV) measurements were taken for the initial three charge–discharge cycles, as shown in Figure 9. The potential window ranged between 0.05 and 3.0 V (vs. Li^+^/Li) at a scan rate of 0.2 mVs^−1^. The CV results for the carbon produced from the thermal decomposition of the phthalocyanine (H_2_PC) have already been discussed elsewhere [49]. The CV results of the VN(TD) electrode (Figure 9) indicate the formation of reduction peaks in the cathodic scan (lithiation) at 2.24–2.4 V, 1.3 V, and 0.8 V, suggesting a potential multiple-step transition [50,51]. The reduction peak at around 0.45–0.8 V (Figure 9) was attributed to the decomposition of the electrolyte and the formation of the solid electrolyte interphase (SEI) [50,52]. These peaks disappear at the first anodic cycle (de-lithiation), while the CV curves for VN indicate good reversibility of Li^+^ storage in the electrodes. Oxidation peaks during the anodic scan (de-lithiation) were observed at 1.3 V, 0.6 V, and 2.49–2.52 V. The conversion mechanism at around 1.3 and 2.52 V occurred during the oxidation and reduction peaks, implying a Li-reversible reaction with VN, which caused the metallic vanadium (V) particles to be embedded in the Li_x_N matrix (Equation (1)). The reaction mechanism of the vanadium nitrides can be seen below:

(1) $$VN+x{Li}^{+}+xe^{-}\to V+{Li}_{x}N$$

**Figure 10:**
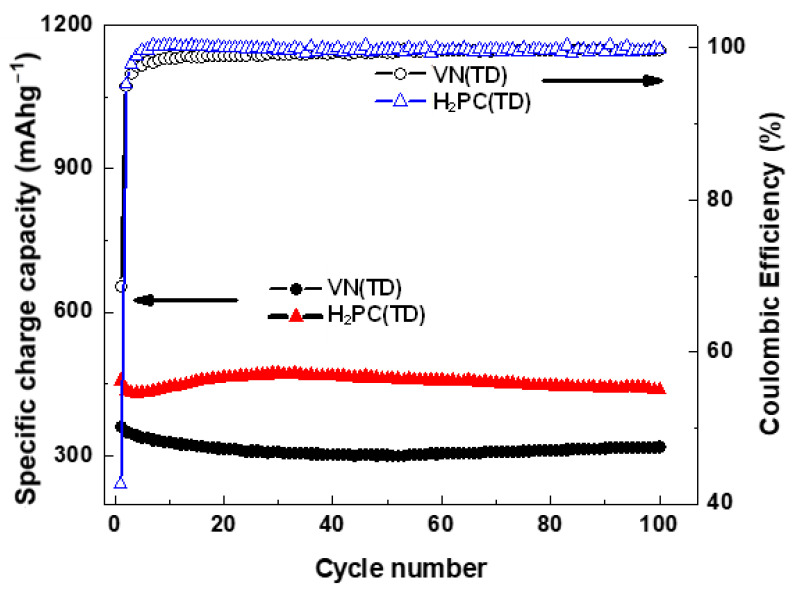
Specific discharge/charge capacity vs. cycle number Coulombic efficiency of VN(TD) and H_2_PC(TD) electrodes after 100 cycles at 100 mAg^−1^.

Figure 10 shows the charge capacity and Coulombic efficiency vs. cycle number of H_2_PC(TD) [49] and VN(TD) electrodes after 100 cycles at a current density of 100 mAg^−1^. The H_2_PC(TD) anode delivered a stable charge capacity, which was higher than that of the VN(TD) electrode. The specific charge capacity of the VN(TD) electrode decreased after a few cycles, slightly increased, and then remained constant at 307 mAh g^−1^ after 100 cycles, indicating a stable electrochemical performance. However, the VN(TD) anode exhibited a larger initial Coulombic efficiency (68.1%) and better capacity retention (98.3% in the second cycle) than the H_2_PC (TD) anode.

## 3. Materials and Methods

### 3.1. Materials Synthesis

All the reagents and solvents used in this work were of analytical grade and used without further purification. The metal-substituted phthalocyanine, the reaction precursor VOPC, was prepared by mixing a 4:1 molar ratio of vanadium (IV) chloride with phthalonitrile in 1-chloronaphthalene and refluxing for 6 h [53,54]. For comparison purposes, non-metalated phthalocyanine (H_2_PC) was also synthesized under the same reaction conditions used for the VOPC synthesis. After cooling to room temperature, the products were collected by filtration, washed with methanol and acetone, and then purified by sublimation. The synthesized VOPC was placed in an alumina boat and loaded into a Thermolyze horizontal tube furnace (model F79330-33-70) (Thermo Fisher Scientific, Waltham, MA, USA) for thermal decomposition, which generated VN in a carbon matrix (VN(TD)). The quartz tube was purged with ultra-high-purity (UHP) nitrogen for 15 min before heating. The temperature of the furnace was increased from room temperature to 750 °C at a rate of 10 °C min^−1^ and maintained for 5 h with a constant flow of UHP N_2_ gas. After 5 h of reaction, the samples were cooled to room temperature naturally while maintaining the nitrogen flow over the sample.

For comparison purposes, VN was also prepared through the sol-gel method (VN(SG)) as follows: 2.25 g of urea was dissolved in 5 mL of ethanol, and 1 mL of vanadium(IV) tetrachloride was added slowly [7]. The gel was poured into a boat and placed in a quartz tube in a Thermolyne horizontal tube furnace (model F79330-33-70) (Thermo Fisher Scientific, Waltham, MA, USA) using the same procedure outlined above.

### 3.2. Characterization

The synthesized compounds were characterized by FTIR, XRD, SEM, TEM, and XPS. The dried materials were ground into a fine powder using a mortar and pestle. FTIR spectra were collected on a Perkin–Elmer Frontier FTIR spectrometer using an attenuated total reflection (ATR) accessory (Perkin Elmer, Waltham, MA, USA). The spectra were recorded in the 4000–650 cm^−1^ range and analyzed using Spectrum software (Version 8.0, Perkin Elmer, Waltham, MA, USA). XRD datasets were collected using a Bruker D2 phaser diffractometer fitted with a cobalt source K_α_ = 1.789 Å and an iron filter (Madison, WI, USA). The diffraction patterns were collected from 10 to 80° in 2θ with a 0.05° step size and 5 s of counting time. The collected diffraction patterns were fitted using the Le Bail fitting procedure in the FullProf Suite (Version 5.10) [55,56] and crystallographic data from the literature [37,38,39].

The morphology of the products was observed using SEM. SEM images were collected using a Sigma VP Carl Zeiss microscope (Carl Zeiss, White Plains, NY, USA) operated with accelerating voltages between 2.0 and 6.0 kV at a working distance of up to 6.5 mm. In addition, the samples were analyzed by EDS mapping EDAX, Octane Super (EDAX, Pleasanton, CA, USA) using TEAM^TM^ V4.5.1 software (eZAF Smart Quant model) to determine the elemental distribution of C, O, N, and V in the synthesized VN (TD) and VN (SG). TEM images of nanoparticles were collected using an FEI Titan G2 60-300 microscope (Thermo Fisher Scientific, Waltham, MA, USA)operated with an accelerating voltage of 200 kV and a scanning TEM convergence semi-angle of 15 mrad. XPS was used to determine the surface chemistry of the samples, employing a Thermo Scientific K-Alpha Photoelectron Spectrometer (Thermo Fisher Scientific, Waltham, MA, USA) using a micro-fused monochromatic Al K-α source with a scan step size of 0.1 eV and an X-ray spot size of 400 µm, analyzed using CASA XPS (Version 2.3.25, Casa Software limited, Teignmouth, UK) software [57]. 

Cyclic voltammetry (CV) of the anodes was evaluated using Li-ion half-cells (CR2032 coin cells, PHD Energy Inc., Georgetown, TX, USA). The electrodes were prepared through a slurry-coating process. The slurry was prepared by mixing 90 wt.% of the active material and 10 wt.% of polyvinylidene fluoride (PVDF) as the binder in dimethylformamide (DMF). The slurry was then coated onto 0.025 mm thick copper foil and left to dry. The electrodes were punched into 0.5″ diameter discs using a precision punch (Nagomi, Hopkins, MN, USA). Lithium metal was used as the counter electrode (0.38 mm thick, Aldrich, St. Louis, MO, USA) with a glass-fiber separator [Separation S240 P25 (Degussa AG, 25 μm)]. The electrolyte was 1 M lithium hexafluorophosphate LiPF_6_, dissolved in 1:1 (*v*/*v*) ethylene carbonate (EC)/dimethyl carbonate (DMC). The cells were assembled in a high-purity argon atmosphere in a glovebox (Mbraun, Stratham, NH, USA). Cyclic voltammetry experiments were performed at a scan rate of 0.2 mV s^−1^ over a voltage range between 0.05 and 3.0 V (Biologic Science Instruments, Seyssinet-Pariset, France). The performance of the electrodes was evaluated by performing galvanostatic charge–discharge experiments using an LANHE battery testing system (CT2001A) with an applied current of 100 mA g^−1^ over 100 cycles with a potential range from 0.05 to 3.0 V.

## 4. Conclusions

An essential aspect of electrode manufacturing is the slurry preparation step, which requires a uniform dispersion of its constituents. The use of a homogeneous carbon-based composite circumvents the step of mixing the dry powdered active species and the conductive material. VOPC is a stable, well-known precursor compound that can be easily and consistently synthesized. In addition, VOPC can be used to synthesize a final product where the VN is embedded in a carbon matrix. The process generates small spherical nanoparticles with an average grain size between 6 and 7 nm, which are uniformly dispersed in the carbon matrix in clusters. The VN(TD) had a specific capacity of 307 mAh g^−1^, which was relatively constant over 100 cycles, indicating good electrochemical stability. The thermal decomposition of metal-substituted phthalocyanines is a facile, rapid, and effective process to produce anode materials for LIBs, offering the advantages of using precursors fabricated by well-known methods with a long shelf life and chemical stability.

## Figures and Tables

**Table 1:** FTIR peak positions and corresponding bond stretching assignments for H_2_PC and VOPC.

H_2_PC Peak Position (cm^−1^)	VOPC Peak Position (cm^−1^)	Assignment
710		C-N [29]
729	724	C-H out of plane deformation [30,31]
762	751	Macrocycle Ring Stretching [29]
778	775	C-N stretching [30]
	801	isoindole stretching coupling N-V-N [29]
839		C-N-C Ring Breathing
872	876	N-H stretching coupling with isoindole deformation [29]
	898	Isoindole deformation with coupling aza stretching [29]
944		
	953	V=O [32]
998	1000	Benzene ring and C=C [29]
1064	1064	C–N stretching in pyrrole vibration [30]
1075	1075	
1091		
1116	1118	C–H in-plane deformation [29,30,31]
1157	1159	C–N in-plane and C–H in-plane [30,33]
1187	1192	isoindole stretching [30]
1275		
1299	1284	C–N in isoindol stretching [30,34]
1324		
1336	1332	C–C in isoindole [30]
1417	1418	isoindole stretching [29]
1437		
1461	1461	C–H in-plane bending [30]
1477	1475	C=N pyrrole [29]
1501	1497	C–H bending in aryl [30]
1523	1521	C–H aryl [30]
1576		
1595	1587	benzene C-C stretching [34]
1610	1607	C–C stretching vibration in pyrrole [30]
2923	2923	C–H stretching [30]
3004	3004	C-H stretching [30]
3050	3050	C–H stretching vibration in the ring [30]
3282		N-H [30]

**Table 2:** EDS analysis of elemental composition.

Sample	Element	Atomic Percentage (%)
VN(SG)	V	33.7
	N	19.2
	O	35.3
	C	11.8
VO(PC)	V	1.5
	N	23.1
	O	0.9
	C	74.5
VN(TD)	V	9.1
	N	10.0
	O	5.9
	C	75.1

**Table 3:** Le Bail fitting results for VOPC, VN synthesized by the sol-gel method, and the thermally decomposed VOPC.

Compound	Space Group	*a* (Å)	*b* (Å)	*c* (Å)	α (°)	β (°)	γ (°)	χ^2^	Reference
α-H_2_PC	C_2_/n	25.76	3.77	23.40	90.00	93.11	90.00	2.07	This work
α-H_2_PC	C_2_/n	26.12	3.80	23.88	90.00	94.16	90.00		[37]
VOPC	P-1	12.06	12.56	8.71	96.20	94.94	68.20	1.67	This work
VOPC	P-1	12.03	12.57	8.69	96.04	94.80	68.20		[38]
VN(TD)	FM-3M	4.13	4.13	4.13	90.00	90.00	90.00	1.02	This work
VN(SG)	FM-3M	4.13	4.13	4.13	90.00	90.00	90.00	1.11	This work
VN	FM-3M	4.13	4.13	4.13	90.00	90.00	90.00		[39]

**Table 4:** Summary of the XPS fittings of the VN(SG), VO(PC), and VN(TD) samples.

Sample	Energy (eV)	V 2p_3/2_	Energy (eV)	V 2p_½_	Energy (eV)	C 1s	Energy (eV)	N 1s	Energy (eV)	O 1s
VN(SG)	513.6	V^3+^-N	521.1	V^3+^-N	284.2	C-C	396.8	N-V^3+^	529.8	O_2ads_
	515.3	VN-O	523.7	VNO	285.4	C=C	399.0	V-N-O	531.2	V-N-O
	516.7	V-O			288.1	C-N/C-O	401.2	Satellite		
VOPC	515.6	V^4+^-N			283.8	C-C	398.3	N-V^4+^	529.9	O_2ads_
	523.4	V^4+^-O			284.9	C=C	398.9	N-C	531.9	V=O
									533.5	OH/H_2_O
VN(TD)	513.2	V^3+^-N	521.0	V^3+^-N	287.9	C-N/C-O	398.1	N-V^3+^	529.9	O_2ads_
	515.0	VN-O	523.4	VN-O	284.3	C-C	400.0	V-N-O	531.9	V-N-O
	516.4	V-O			285.5	C=C			533.3	OH/H_2_O
					287.4	C-N				

## Data Availability

The data presented in this study are available on request from the corresponding author.
